# Cognitive Remediation for Schizophrenia with Focus on NEAR

**DOI:** 10.3389/fpsyt.2017.00304

**Published:** 2018-01-17

**Authors:** Tamiko Mogami

**Affiliations:** ^1^Faculty of Medicine, Tottori University, Yonago, Japan

**Keywords:** cognition, cognitive remediation, schizophrenia, motivation, intervention

Cognitive remediation or CR is increasingly gaining attention in the field of psychiatry. CR is still a relatively new intervention method for persistent and severe psychiatric illness such as schizophrenia. This article purports to provide general overview of CR and refers to NEAR as its specific example, with the hope that the article will be of help to solicit interest of professionals in psychiatry.

Cognitive remediation has been reported to improve cognition such as attention (1) processing speed (2), immediate learning and memory (1), verbal working memory (3), and problem-solving (4).

In symptomatology of schizophrenia, symptoms are traditionally described in the three groups; positive symptoms, negative symptoms, and thought disorders. More recent literature describing the major symptom groups of schizophrenia might mention positive symptoms, negative symptoms, and cognitive dysfunction. Prevalence of cognitive dysfunction in schizophrenia has been discussed (5). It is observed in more than 80% of people with schizophrenia (5). Cognitive dysfunction occurs as early as the first episode of the illness and persists through the late episodes. Its commonality and severity demand targeting intervention. Severity of the dysfunction varies depending on specific domain of cognition, and most common cognitive domains that are known to be seriously impaired include verbal learning, executive functions, vigilance, motor speed, and verbal fluency (5).

Cognitive dysfunction has been associated with social functioning, such as work, education, independent living, and leisure. More specifically, a study has pointed out that cognition such as vigilance, working memory, verbal memory, and executive functions are positively associated with functional outcomes including social function and occupational function (6). Another study showed that cognition such as working memory, attention, perceptual processing, verbal memory, and processing speed accounted for 52% of functional outcome variance including returning to work or school (7).

While cognitive dysfunction may be considered as a primary feature of schizophrenia, some myths may warrant clarification (5). Cognitive dysfunction is not caused by positive symptoms (5). Cognitive dysfunction is not primarily caused by medications, although there are some exceptions. Although cognitive dysfunction is associated with negative symptoms, it is not caused by negative symptoms. Cognitive dysfunction is not a reflection of overall cognitive decline.

Cognitive remediation was designed as a method of intervention to address cognitive dysfunction. CR may be considered as one of the rehabilitation tools for schizophrenia. As a relatively new method of treatment in psychiatry in Japan, CR is sometimes misunderstood as a choice of intervention for dementia, or as a subtype of cognitive-behavioral therapy. CR is unlike other treatment methods in that it targets neurocognition directly.

Cognitive remediation has several approaches and each has different characteristics, such as use of computer (8), inclusion of employment training (9), focus on a particular aspect such as social cognition (10), or emphasis of motivation (8). CR approaches differ from each other by training focus such as drill practice or strategy, by training model such as rehabilitation or compensation, and by perspectives on hierarchical order of cognition task such as bottom-up perspective or top-down perspective.

When new psychosocial intervention approach such as CR is introduced, its dissemination is often dependent upon practical issues. Effectiveness of CR is tested in a controlled laboratory setting, which is difficult to replicate in a real-world, clinical setting (11). Ease of delivery, clinician training, and clinician supervision, appear to affect which method of CR is selected by a site. Structured or manualized method of CR is generally preferred, as the presence of a structure in a psychosocial intervention tends to improve treatment or implementation fidelity (11).

Delivery of CR has challenges in some settings, such as non-English speaking countries. When CR uses computer software programs, finding appropriate ones may pose difficulty, as the same software programs mentioned in CR treatment manuals may not be available or usable due to language issues. Likewise health insurance system differs depending on a country where CR is delivered, and affects if and how CR may be offered.

NEAR is a form of CR and stands for Neurocognitive and Educational Approach to (Cognitive) Remediation. NEAR was developed by Medalia and her colleagues (12). In NEAR, two computerized sessions and one verbal session a week are typically offered. Sessions are offered in a small group format, which facilitates interpersonal aspect of rehabilitation. NEAR is based on educational principles and emphasizes intrinsic motivation (IM). NEAR has been effective in improving cognition and social functioning (13). The improvement has been maintained as long as 4-month follow-up (13).

NEAR is based on learning principles and views CR as a form of education (14), as CR asks individuals to do something in a new manner. In a traditional learning model, individuals’ ability determined the learning outcome, leaving small room for change by intervention. However, in a more recent learning model, individuals’ cognitive ability, motivation, and mode of instruction all affect one another, determining learning outcomes (15).

In NEAR, intake interview is conducted prior to a patient’s enrollment in a group. Cognition is assessed prior to the beginning of NEAR, and relevant background information is gathered such as educational history, employment history, learning styles, and rehabilitation goals. In some instances, a neuropsychological testing may be conducted to measure cognition. Rehabilitation goals may be in the domains of education, employment, independent living, or leisure, and it is desirable that these goals are specific. Specific rehabilitation goals may be used to illustrate how mastering a specific cognitive task is helpful in real life.

People with severe psychiatric illness such as schizophrenia tend to have impairment in motivation, as noted in avolition or anhedonia. IM has been studied as a mediating factor between cognition and social functioning (16). The need for intervention for schizophrenia to enhance IM has been shown (17). It has been demonstrated that CR that enhances IM, compared to CR which does not, improves the sense of competence, and cognition more significantly (8).

There is a range of motivation from rather external to IM (18). Motivation has been linked to behaviors, and an externally motivated individual may engage in a particular behavior due to external forces or encouragement such as teacher, medical staff, a high test score, or monetary reward. An intrinsically motivated individual may engage in a particular behavior because he/she may regard the behavior as interesting, enjoyable, valuable, or at which they are competent. Intrinsically motivated individuals may engage in a behavior when they consider that they are autonomously choosing the behavior, and it is not forced upon them.

Intrinsic motivation theoretically is based on self-determination theory or SDT (18). SDT posits the three needs that are associated with IM: competence, (personal) relatedness, and autonomy. SDT shows how behavior’s regulatory style differs from external regulation to intrinsic regulation (IM). In NEAR, patients are encouraged to regulate behaviors in multiple ways (i.e., external vs. internal), in cognitive tasks and intervention techniques; it was shown that intervention that incorporated SDT improved cognition larger compared to intervention that did not (8).

Analogous to learning in an educational setting, people engage in tasks based on expectancy-value theory in psychiatric rehabilitation (19). It is said that people tend to engage in new behavior (and for most patients in CR, working on cognitive tasks is new behavior) when they feel in control, autonomous, competent, efficacious, and when they find the behavior interesting or meaningful. To address these issues and to ultimately enhance IM so that people with psychiatric illness can develop autonomous help-seeking behaviors, NEAR incorporates motivation enhancing components in cognitive tasks and intervention techniques.

NEAR uses both restorative and compensatory approaches. Intervention techniques include errorless learning, shaping, and prompting (12). Errorless learning introduces a task at a certain difficulty level only after easier levels are mastered, so that chances for making errors are limited. Shaping enables goal attainment as it presents with achievable, non-threatening, small goals. In prompting, facilitator may suggest what behaviors to take without giving the answers. The interventions are different from providing the answer, as it gives the patient a chance to solve the task themselves.

The techniques make it possible for patients to engage in tasks autonomously. NEAR facilitators act like a coach for athletes using these techniques: while they may show how to solve a problem, they typically do not provide the answers. NEAR cognitive tasks may be contextualized in life so that trained cognition may be easily transferred to real life situations.

NEAR facilitates a verbal session in addition to a cognitive session. Verbal session is also referred to as bridging session, as it bridges cognitive tasks to real life cognition, or life goals such as vocational goal. Bridging session purports to transfer of cognitive skills trained in cognitive sessions to real-life situations. Bridging session may take a form of a discussion that allows participants present or learn about types of cognitive tasks they are working on, or a group game which allows participants to use cognitive skills they are training, or a group session with emphasis on communication. For instance, a patient may recall a memory task from a cognitive session and make a connection to real life by stating that he/she must memorize item placements and pricing at work.

Cognitive remediation outcome has been reported from meta-analyses. Interested readers are suggested to refer to meta-analyses that compare the outcomes of different CR methods (19, 20). A meta-analysis of 26 CR outcome studies revealed improvement of cognition with medium effect size of 0.41 (21). NEAR has improved cognition such as attention (8), processing speed (21, 22), immediate learning and memory (1), and delayed verbal memory (22). NEAR outcome studies have been conducted at a variety of settings such as outpatient psychiatric facilities (1, 13), inpatient psychiatric units (4), intensive psychiatric rehabilitation (23), supportive housing facility (24), and its controlled trials have been conducted (3, 13): in these studies, computer sessions were provided once or twice a week, and the duration of the treatment differed from 4 to 15 weeks. Longer treatment duration is recommended to achieve clinically significant change (12). Outcome studies do not clarify whether verbal session was conducted.

Cognitive remediation has been known to be most effective when combined with a comprehensive psychiatric rehabilitation (25), and when drill-and-practice coaching is combined with strategy coaching (21). CR may be provided as a part of a more comprehensive psychiatric rehabilitation, such as psychiatric day treatment. Neurocognition may be trained in CR while the skills connected to the same domain may be practiced in other programs such as farming, cooking, or athletic sports. In a CR program such as NEAR, patients have a chance to practice certain skill repeatedly, just as one might practice striking a baseball, while they also have a chance to strategize their approach to a cognitive task.

Patient factors known to be associated with CR outcomes include baseline cognition, clinical stability (26), motivation (25), and phase of illness (27). Severity of positive or negative symptoms, provided pharmacological treatment is provided, does not seem to affect their eligibility for CR. It seems to stand for a reason that a strong motivation as measured by attendance rate was positively associated with improvement in cognition (25). Motivational interviewing has also been conducted prior to patients’ enrollment in CR (28). CR outcome studies divided the patients depending on the phase of illness consisting of early course and chronic phase. Early-course patients, when compared to chronic patients, mostly showed larger improvement in cognition (27), but the reasoning behind the difference is yet to be determined.

Limitations of CR outcome studies are listed here, providing their future directions. Overlap of treatment providers and evaluators may crowd the outcome evaluation. Variability of control conditions makes it difficult to determine the effectiveness of intervention among different trials. CR treatment fidelity is not clearly established in some studies, making it difficult to know specificity of how CR was provided. Variability of outcome scales makes it difficult to compare effectiveness of CR among different trials. Providing a specific manner which ascertains treatment fidelity is needed to improve the quality of CR outcome studies, while also enhancing optimal CR in clinical settings.

## Author Contributions

The author conceptualized and wrote this manuscript.

## Conflict of Interest Statement

The author declares that the research was conducted in the absence of any commercial or financial relationships that could be construed as a potential conflict of interest.
